# Age-Related Differences in Alcohol Intake and Control Over Alcohol Seeking in Rats

**DOI:** 10.3389/fpsyt.2018.00419

**Published:** 2018-09-03

**Authors:** Maaike Labots, Janna Cousijn, Linda A. Jolink, J. Leon Kenemans, Louk J. M. J. Vanderschuren, Heidi M. B. Lesscher

**Affiliations:** ^1^Department of Animals in Science and Society, Division of Behavioural Neuroscience, Faculty of Veterinary Medicine, Utrecht University, Utrecht, Netherlands; ^2^ADAPT-Lab, Department of Psychology, University of Amsterdam, Amsterdam, Netherlands; ^3^Amsterdam Brain and Cognition (ABC), University of Amsterdam, Amsterdam, Netherlands; ^4^Department of Experimental Psychology, Helmholtz Research Institute, Utrecht University, Utrecht, Netherlands

**Keywords:** alcohol, loss of control, adolescence, addiction-like behavior, conditioned suppression, individual differences, rats

## Abstract

Alcohol use disorder (AUD) is characterized by excessive and persistent alcohol use, despite adverse consequences. AUD often originates during adolescence, as do other substance use disorders. However, despite periods of excessive alcohol intake, many adolescents reduce their alcohol use by early adulthood. Brain development, social context, personality traits, and genetic makeup are thought to play an important role in these age-dependent fluctuations in alcohol use. However, studies that directly investigate age-related differences in the effects of alcohol exposure on brain and behavior are sparse. Therefore, to better understand the relationship between adolescent alcohol consumption and AUD-like behavior, this study compared the degree of control over alcohol seeking in rats that differed in terms of age of onset of alcohol drinking and in their level of alcohol consumption. We hypothesized that control over alcohol seeking is more prominent in adolescent-onset rats than in adult-onset rats, and that control over alcohol seeking is related to the consumed amount of alcohol. To test this hypothesis, alcohol seeking in the presence of a conditioned aversive stimulus was assessed after 2 months of intermittent alcohol access (IAA) in rats that consumed alcohol from postnatal day 42 (adolescence) or day 77 (adulthood). The rats were subdivided into low (LD), medium (MD), or high (HD) alcohol drinking rats, in order to assess the impact of the extent of alcohol intake on control over alcohol seeking. The adolescent-onset animals consumed slightly, but significantly less alcohol compared to the adult-onset rats. In adult-onset rats, we found that conditioned suppression of alcohol seeking, i.e., reduction of alcohol seeking by presentation of a conditioned aversive stimulus, was most pronounced in LD. By contrast, in the adolescent-onset rats, MD and HD showed increased alcohol seeking compared to LD, which was suppressed by conditioned aversive stimuli. Taken together, these findings reveal a complex relationship between the age of onset and level of alcohol intake with control over alcohol seeking, whereby adolescent rats consume less alcohol than adults. In adult rats, control over alcohol seeking is negatively related to preceding levels of alcohol intake. By contrast, adolescent rats appear to retain control over alcohol seeking, even after a history of high levels of alcohol intake.

## Introduction

Alcohol is among the most widely used substances of abuse and alcohol use disorder (AUD) remains an enormous public health problem, affecting over 76 million people worldwide (1–3). AUD is characterized by excessive alcohol use and loss of control over alcohol intake, signified by persistent alcohol use despite adverse consequences (4). Considering that only a minority of the individuals that use alcohol develop an AUD, it is important to understand the factors that determine the transition from recreational and controlled, to compulsive and uncontrolled alcohol use. AUD often originates during adolescence and young adulthood, as do other substance use disorders. Adolescent alcohol use has been shown to be detrimental for brain development [for review see (5)]. At the same time, risk taking behavior during adolescence, which is expressed, amongst others, through substance use, is increasingly considered to be important for adolescents to mature and develop a degree of behavioral flexibility, that is necessary to thrive as adults [e.g., (6)]. Importantly, even after periods of excessive alcohol use, many adolescents show a reduction in their alcohol use by early adulthood (7). Brain development, social context, personality traits and genetic makeup are thought to play an important role in these age-dependent fluctuations in alcohol use (8).

Human literature suggests that the age of onset of alcohol use is associated with the risk to develop an AUD later in life. Population based studies have suggested that the earlier the onset of alcohol consumption, the greater the likelihood to develop heavy alcohol use, alcohol-related problems and an AUD (9–16). However, age of first alcohol use is not specifically associated with AUD but also with the development of other mental disorders (17). Moreover, recent systematic reviews suggest that the evidence for the association between age of first drink and AUD is less consistent and may be driven by confounding factors, such as socioeconomic status, a history of alcohol problems in the family or preceding mental health problems that may have contributed to the early onset of drinking and the risk for AUD (18, 19). The impact of adolescent alcohol exposure on alcohol consumption and other reward-related behaviors in adulthood has also been addressed in animal studies that have yielded a picture that is far from straightforward. For example, it has been shown that adolescent alcohol exposure enhances alcohol consumption and operant self-administration of alcohol in adulthood in rats and mice (20–24). However, other studies have found that adolescent alcohol exposure does not affect alcohol intake in adulthood (25), or only upon vapor-induced dependence (26) or alcohol deprivation (27). Moreover, direct comparison of adolescent vs. adult onset of alcohol intake suggests that alcohol intake is not different in animals with an adolescent onset of alcohol consumption, compared to rats that started to consume alcohol in adulthood (28, 29). With regard to the associative structure of alcohol use, Serlin and Torregrossa (30) have shown that rats that started to self-administer alcohol in adolescence were more goal-directed in their alcohol seeking (i.e., alcohol seeking was sensitive to the subjective value of alcohol) compared to adult-onset animals, that more readily developed habitual alcohol seeking. On the other hand, alcohol exposure during adolescence has been shown to enhance risky behavior in adulthood [(31–33); but see (34)], as well as the responsiveness to reward-predictive cues (35, 36). Importantly, the studies described above used different, often overlapping age ranges, making it unlikely that discrepancies in the findings can simply be explained by age differences, such as between early or late adolescence (37, 38).

Taken together, there is evidence that adolescent onset of alcohol use alters behavioral traits related to AUD, although the differential impact of adolescent- vs. adult-onset alcohol use on the development of AUD-like behavior remains largely unexplored. The aim of this study was therefore to compare the influence of age of onset of alcohol consumption on control over alcohol seeking in rats. To address this, alcohol consumption and control over alcohol seeking was determined in rats that initiated alcohol consumption during adolescence or adulthood. In order to also take individual variation in alcohol consumption into account, we used outbred Lister Hooded rats and an intermittent alcohol consumption paradigm, similar to previous studies in which we demonstrated individual differences in alcohol consumption that are associated with loss of control over alcohol seeking (39, 40). We hypothesized that control over alcohol seeking in rats would be related to the level of alcohol intake (39, 40), that control over alcohol seeking would be more prominent in adolescent-onset rats than in adult-onset rats and that the age related difference in control over alcohol seeking would be especially pronounced in high alcohol consuming animals.

## Materials and methods

### Animals

Male Lister Hooded rats (Charles River, Sulzfeld, Germany) arrived either at postnatal day (PND) 35 (adolescent, *N* = 84) or 70 (adult, *N* = 84) in the facility and were housed in type III Makrolon cages with matching type III-S lids under controlled conditions, with a reversed 12 h light/dark cycle (lights off 7.00 a.m.) and *ad libitum* access to water and chow. The cages were enriched with a homemade square PVC shelter and paper tissues. The rats were acclimatized to the facility for 1 week upon arrival. Thereafter, the rats were housed individually and they were weighed and handled at least once per week. All experiments were approved by the Animal Ethics Committee of Utrecht University and conducted in agreement with Dutch laws (Wet op de dierproeven, 2014) and European regulations (Guideline 86/609/EEC).

### Voluntary home-cage alcohol consumption

The rats were allowed to consume alcohol according to an intermittent alcohol access (IAA) schedule. The rats were given access to 20% (v/v) alcohol (Klinipath, The Netherlands) and water in a two-bottle choice setup in the home-cage with IAA for 3 days a week (Monday–Wednesday–Friday). For voluntary alcohol consumption, transparent 250 ml bottles (Techniplast, Germany) were used and the position of the alcohol and water bottle was switched between sessions. On alcohol-free days the rats had only one 500 ml bottle, filled with water. The adolescent rats started to consume alcohol from PND 42, while the adult animals started with voluntary alcohol consumption from PND 77. We chose to start adolescent voluntary alcohol consumption at PND 42 because we wanted to capture the developmental period starting at puberty, which in male rats is around PND 40 (41). Moreover, we wanted to avoid social isolation during the period in life when rats show most social play behavior (i.e., PND 21-42), since we and others have previously shown that animals deprived from social play show increased sensitivity to the rewarding properties of substances of abuse (42–44). We therefore started the drinking study, which requires the rats to be housed individually, from PND 42. During the first month of IAA, alcohol was presented for 7 h/day (9.00 a.m. until 16.00 p.m.) and access to alcohol was extended to 24 h/day in the second month of the experiment [see (39)]. Alcohol intake (g/kg) and preference (% volume of alcohol over water) were calculated per rat per session and subsequently averaged per week. After 2 months of voluntary home-cage alcohol consumption, the rats were classified into low (LD), medium (MD), or high (HD) alcohol drinking groups based on their average home cage alcohol intake per week across the 2 months using a tertile split of the population.

### Operant alcohol self-administration

After 2 months of voluntary home-cage alcohol consumption, operant responding for alcohol in the presence of an aversive conditioned stimulus, i.e., conditioned suppression of alcohol seeking, was assessed in order to investigate the influence of adolescent-onset vs. adult-onset alcohol exposure on control over alcohol use.

#### Operant training

The rats were trained to lever press for alcohol in operant conditioning chambers (Med-Associates, USA) 3 days/week (Monday–Wednesday–Friday) as previously described (40). The position of the active and inactive levers was counterbalanced between rats. Upon meeting the response requirement, the dipper cup containing alcohol (0.1 ml, 20% v/v) was raised, the cue light above the active lever was illuminated and the house light was switched off. Ten seconds after a head entry into the magazine, access to alcohol was terminated, the cue light was turned off and 5 s later a new trial started. Pressing the inactive lever was recorded, but had no programmed consequences. The rats were initially trained to respond for alcohol under a Fixed Ratio 1 (FR1) schedule of reinforcement for three sessions. Thereafter, the rats were trained under a random interval (RI) schedule of reinforcement, whereby the first active lever press initiated a RI during which lever pressing was recorded but was without consequences. After the RI had elapsed, the first active lever press resulted in the delivery of alcohol. The rats were trained in 30 min RI sessions with increasing RI durations (3 sessions with a 5 s RI, followed by 3 sessions with a 15 s RI, 2–3 sessions with a 30 s RI and then 2–3 sessions with a 60 s RI). Finally, the rats were trained in five 60 min RI 120 s sessions. Thereafter, the data were analyzed to verify stable responding under the RI120 schedule of reinforcement, i.e., <25% variation in active responses during the last three RI 120 s sessions. To determine the stability of alcohol seeking, only the first 15 min of the last three RI 120 s sessions were considered to match the duration of the conditioned suppression tests (see below). Experimental events and data recording were controlled using MED-PC software.

#### Conditioned suppression of alcohol seeking

After the RI training was completed, conditioned suppression was performed according to previously described procedures (40, 45–47). Thus, the LD, MD, or HD rats were assigned to subgroups that either underwent fear conditioning, with conditioned stimulus - footshock pairings (CS+), or underwent control conditioning (CS–). A CS-shock conditioning session started with a 5 min period in which only the house light was illuminated, followed by two periods of 10 min during which a 85 dB, 2,900 Hz tone (separated by a 10 min inter-trial-interval) was constantly presented. During the 10 min tone presentations, 10 unpredictable footshocks (1 s duration) were delivered, resulting in a total of 20 shocks for each CS+ rat. The conditioning session was completed after a final 5 min period without tone presentation. Rats in the CS– control group underwent the same procedure, but without footshocks. To ensure equal mean seeking rates for CS+ and CS– groups prior to conditioning, the rats' mean seeking responses per minute during the first 15 min of the last three RI 120 s sessions, chosen to align with the duration of the conditioned suppression tests, were used to assign the rats to the respective CS- and CS+ groups. From the adolescent-onset rats, a total of 14 LD, 14 MD, and 14 HD underwent control conditioning (CS–) and 14 LD, 14 MD, and 14 HD were fear-conditioned with 0.4 mA footshocks. In the adult group, 3 animals were lost due health problems prior to conditioned suppression, leaving a total of 14 LD, 13 MD, and 14 HD in the CS– groups and a total of 14 LD, 13 MD, and 13 HD in the CS+ groups, respectively. To prevent association of fear conditioning with the self-administration environment, acquisition of the CS-shock association was established in conditioning chambers that were physically different from operant self-administration (SA) chambers, in that the conditioning chamber had a curved wall with 5 nose poke holes and no levers and stimulus lights. To facilitate CS-shock associations, rather than context-shock associations, the rats were habituated to the conditioning chambers in three 30 min sessions on separate days prior to conditioning.

After conditioning, the rats received two additional RI 120 s training sessions. Subsequently, conditioned suppression of alcohol-seeking behavior was assessed in the SA chambers. The house light was illuminated and 2 min after the start of the session, the levers were extended for the remaining 12 min of the test. Alcohol seeking during the conditioned suppression test was examined in extinction, i.e., responding on the levers was recorded, but had no programmed consequences. To avoid altered responding due to the lack of (smell of) alcohol in the SA chamber, the cup containing 20% alcohol (v/v) was present underneath the liquid dipper but the rats did not have access to the solution. Three 2-min intervals in which the tone CS was presented (CS-ON interval) were alternated with 2-min intervals where the tone CS was absent (CS-OFF interval). Active lever presses and latency to the first lever press were recorded and compared for CS– and CS+ subgroups as a measure of control over alcohol seeking.

#### Conditioned freezing

One week after completion of the conditioned suppression test, the rats underwent fear conditioning (CS+) or control conditioning (CS–); the rats were assigned to the same group as for conditioned suppression of alcohol seeking. Fear conditioning procedures were similar to those described in the previous section. After 24 h, the rats were placed in the conditioning chamber: 2 min without the CS+ tone and subsequently 2 min with the CS+ tone. The frequency and duration of freezing behavior, defined as the absence of any movement other than breathing (40, 48–50), was scored from DVD-taped behavior using Observer software (Noldus, Wageningen, NL) by an observer blind to age, alcohol drinking group and conditioning history.

### Statistics

To compare the level of alcohol consumption in the two age groups and the subgroups of low, medium and high alcohol drinking rats, the alcohol intake (g/kg), preference (% choice for alcohol vs. water), and total volume of fluid intake (ml/kg) data were analyzed by three-way repeated measures ANOVA with month (1st- 7 h and 2nd- 24 h) as the within-subjects factor and age group (adolescent and adult onset) and group (LD, MD, HD) as the between-subjects factors. Moreover, to test our hypotheses that control over alcohol seeking in rats would be more prominent in adolescent-onset than in adult-onset rats and that the age related differences in control over alcohol seeking would be more pronounced in the high alcohol consuming animals, the conditioned suppression data were analyzed by four-way repeated measures ANOVA with tone (ON/OFF) and interval (3 repetitions for ON/OFF) as the within-subjects factors and age group (adolescent and adult onset), CS (CS+/CS–) and group (LD, MD, HD) as the between-subjects factors. Mauchly's test of sphericity was used to test if variances of the differences between treatment levels were equal. If the assumption of sphericity was violated, degrees of freedom were corrected using Huynh-Feldt estimates of sphericity to more conservative values; corrected degrees of freedom are presented rounded to the nearest integer. When appropriate, *post-hoc* analyses were conducted using multiple LSD pairwise comparisons. Each parameter was tested for normality with a Kolmogorov-Smirnov test. In case the behavioral parameters were not normally distributed (i.e., the latency data from the conditioned suppression test), data was log transformed prior to statistical analyses, which resulted in normal distribution of the data. Statistical analyses were conducted using IBM SPSS Statistics for Windows, version 22.0 (IBM Corp., Armonk, N.Y. USA). The threshold for statistical significance was set at *p* < 0.05. All data are presented as mean + SEM.

## Results

### Alcohol consumption

**Figure 1:**
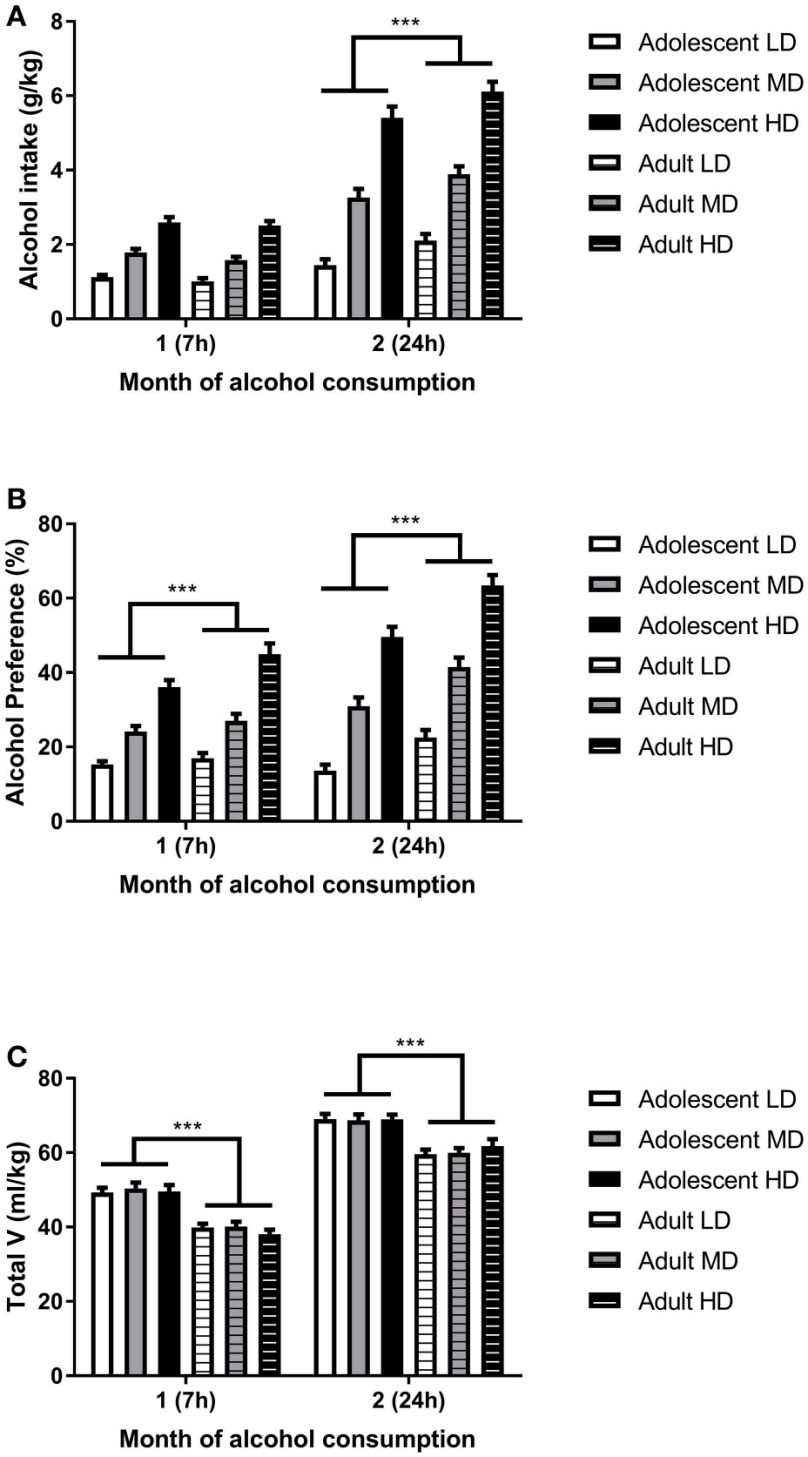
Alcohol intake in adolescent onset and adulthood onset rats. Depicted are alcohol intake **(A)**, alcohol preference **(B)**, and total volume (V) of fluid consumed **(C)** for selected low drinkers (LD), medium drinkers (MD), and high drinkers (HD) from both age groups. The data are shown as mean + SEM. ***Significant overall difference between adolescent-onset and adult-onset rats, independent of drinking group by *post-hoc* pairwise comparison, (*P* < 0.001).

Analysis of the alcohol intake data (Figure 1A) revealed that, in line with previous studies (36, 39, 40, 44), alcohol intake was higher in the second month of the experiment [*F*_(1, 161)month_ = 471, *P* < 0.001], when access to alcohol was extended from 7 to 24 h. This escalation of alcohol intake during the second month of access was dependent on the age of onset, as is evident from a significant month × age group interaction [*F*_(1, 161)_ = 20.1, *P* < 0.001]; escalation of alcohol intake was more pronounced in adult-onset rats. Overall, the group that started to consume alcohol as adults consumed more alcohol compared to the rats that started to consume alcohol during adolescence [*F*_(1, 161) age group_ = 5.4, *P* < 0.05]. *Post-hoc* pairwise comparisons showed that the difference in alcohol intake between the age groups was only significant for the second month, when the rats had 24 h access to alcohol. The increase in alcohol intake in the second month was also dependent on the level of alcohol consumption of the individual animals [F_(2, 161) month × group_ = 65.7, *P* < 0.001], in that HD showed a greater increment compared to LD and MD. Further, the selected HD overall consumed more alcohol than the LD and MD [*F*_(2, 161) group_ = 195.1, *P* < 0.001], independent of the age of onset [*F*_(2, 161) age group × group_ = 0.07, N.S.]. Similar effects were observed for the preference for alcohol over water (Figure 1B) and overall, adolescent rats consumed more fluids when compared to their adult counterparts (Figure 1C).

**Figure 2:**
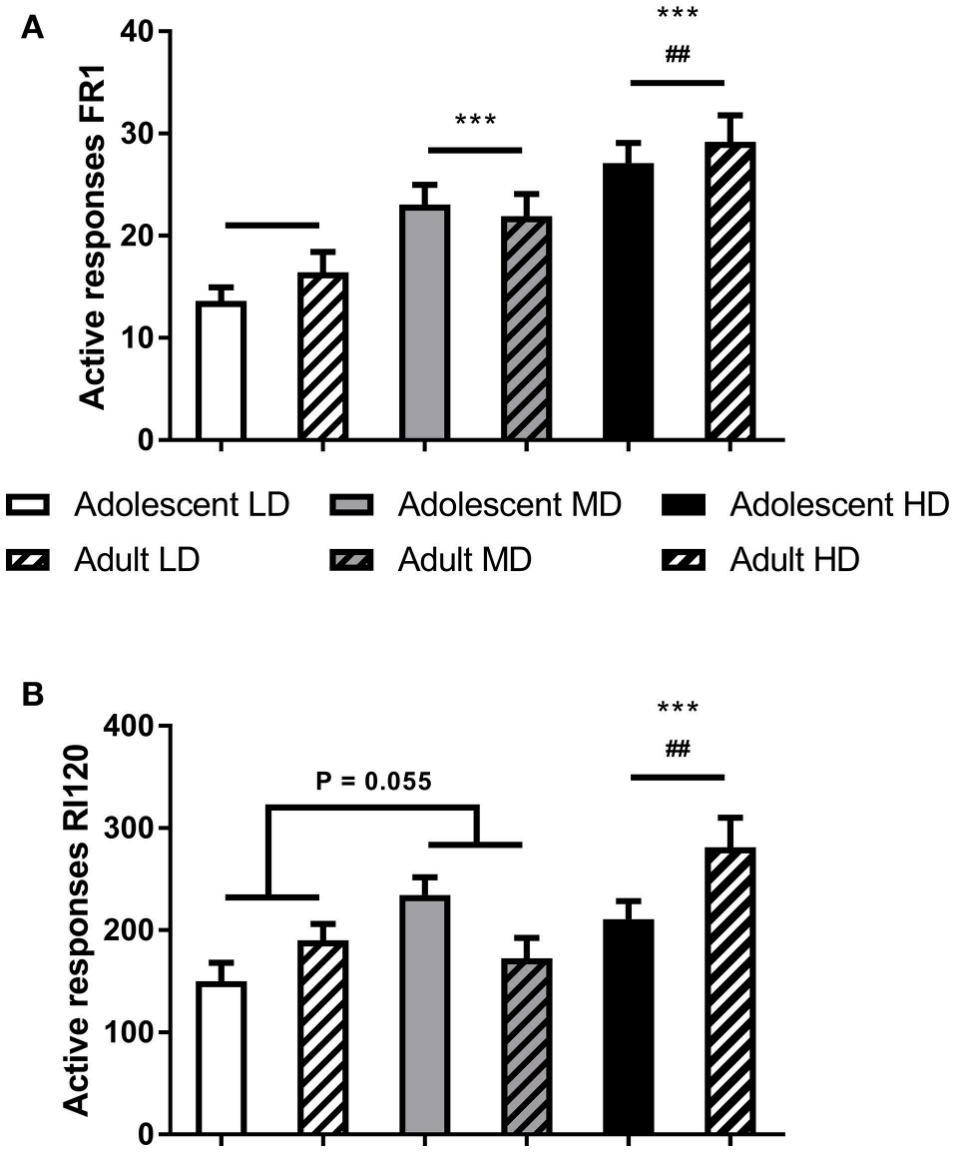
Responding for alcohol under FR1 **(A)** and RI120 **(B)** schedules of reinforcement. Shown are the average number of active responses during the (last) three sessions of training on the respective schedules for adolescent-onset and adult-onset low (LD), medium (MD), and high alcohol drinking (HD) rats after 8 weeks of intermittent alcohol consumption. Data are presented as mean + SEM active responses. ***MD and HD made more active responses compared to LD, independent of age group (*post-hoc* pairwise comparisons, *P* < 0.001) and ^*##*^HD made more active responses than MD, independent of age group (*post-hoc* pairwise comparisons, *P* < 0.01).

Importantly, the groups remained different during operant alcohol self-administration. Analysis of the number of active responses confirmed overall effects of drinking group during the initial FR1 training sessions [Figure 2A; *F*_(2, 166) group_ = 21.0, *P* < 0.001] and during the final RI 120 s sessions [Figure 2B; *F*_(2, 166) group_ = 11.7, *P* < 0.001], with a trend for an age group effect on the number of active responses during RI120 training sessions [*F*_(1, 166) age group_ = 3.3, *P* = 0.07]. *Post-hoc* pairwise comparisons revealed that MD made more responses for alcohol than LD and HD made more responses for alcohol than LD and MD.

### Conditioned suppression of alcohol seeking

**Figure 3:**
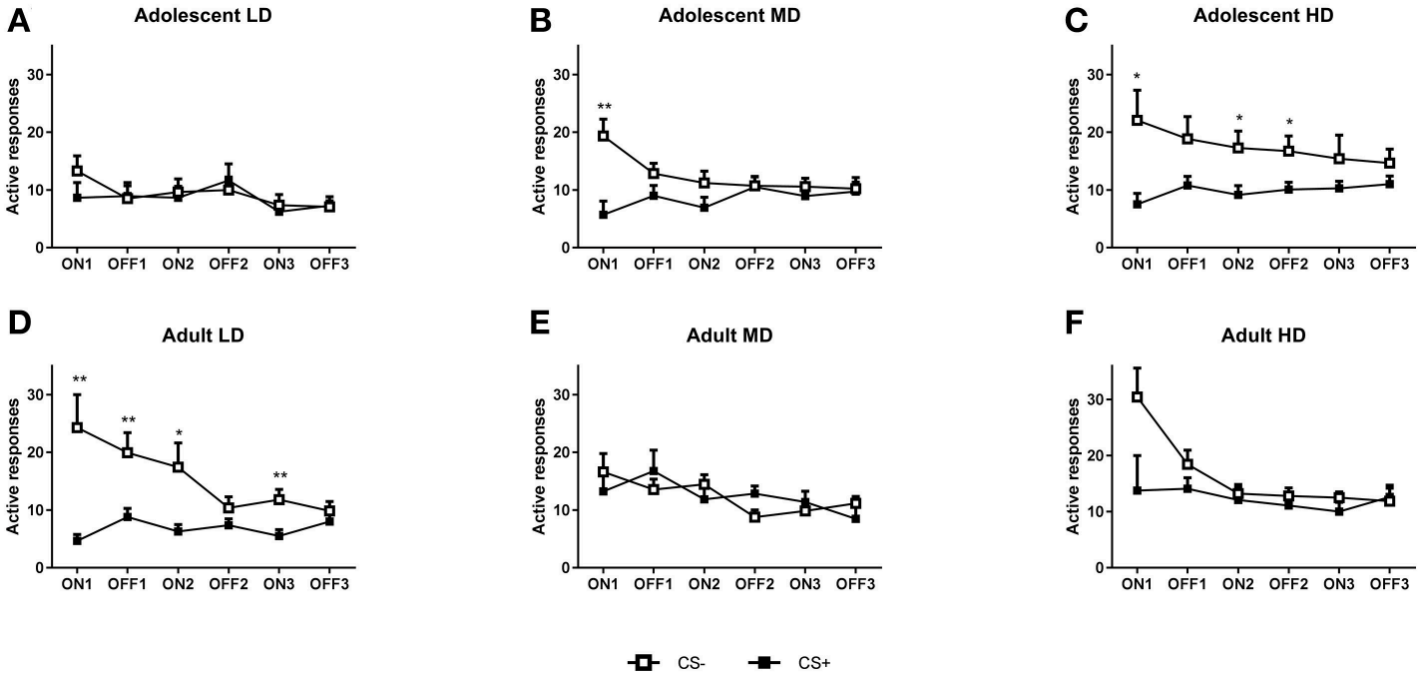
Conditioned suppression of alcohol seeking in CS– and CS+ groups of adolescent-onset and adult-onset low (LD), medium (MD), and high alcohol drinking (HD) rats after 8 weeks of intermittent alcohol consumption. In **(A–C)**, the number of active responses during consecutive CS ON and CS OFF intervals are shown for adolescent-onset LD, MD, and HD, respectively. In **(D–F)**, the number of active responses during CS ON and CS OFF intervals are shown for adult-onset LD, MD, and HD, respectively. Data are presented as mean + SEM active responses, binned in 2 min intervals. Significant differences between CS– and CS+ groups are indicated by * and ** (*post-hoc* pairwise comparisons, *P* < 0.05 and *P* < 0.01, respectively).

**Figure 4:**
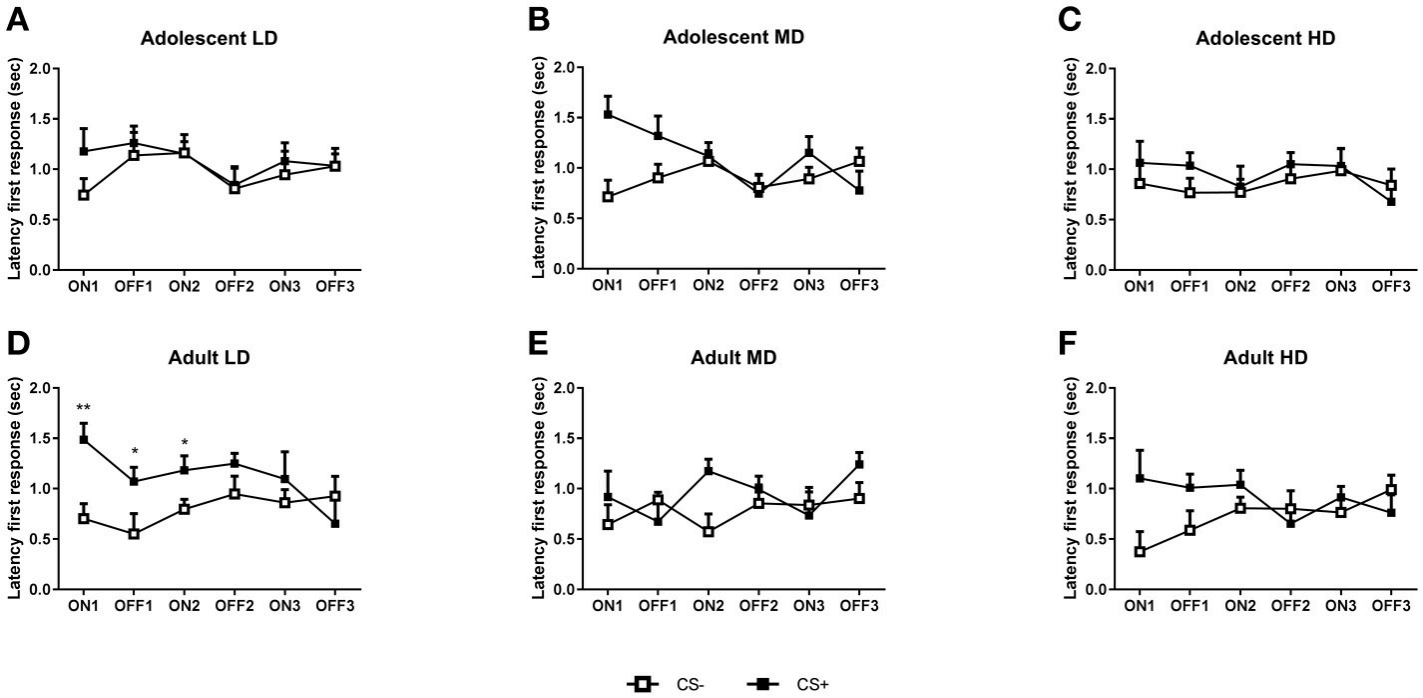
Conditioned suppression of alcohol seeking in CS– and CS+ groups of adult-onset and adolescent-onset low (LD), medium (MD), and high alcohol drinking (HD) rats after 8 weeks of intermittent alcohol consumption. In **(A–C)**, the latency to the first active response during consecutive CS ON and CS OFF intervals are shown for adolescent-onset LD, MD, and HD, respectively. In **(D–F)**, the latencies to respond during CS ON and CS OFF intervals are shown for adult-onset LD, MD, and HD, respectively. Data are presented as mean + SEM active responses, binned in 2 min intervals. Significant differences between CS– and CS+ groups are indicated by * and** (*post-hoc* pairwise comparisons, *P* < 0.05 and *P* < 0.01, respectively).

For the analysis of conditioned suppression of alcohol seeking (Figures 3, 4), the LD, MD, and HD groups for both the adolescent- and adult-onset groups were further subdivided into CS– and CS+ animals (*N* = 13–14). Analysis of the number of active responses for the CS– control groups by age revealed that the adolescent-onset groups tend to differ in their responding during the conditioned suppression test [*F*_(2, 39) group_ = 3.21, *P* = 0.051]. *Post-hoc* pairwise comparisons revealed that adolescent-onset LD made fewer responses compared to HD rats, but LD were not different from MD that in turn did not significantly differ from the HD. The differences in active responses between the groups were not dependent on tone presentation [*F*_(2, 39) tone × group_ = 0.17, N.S.; *F*_(4, 78)tone × interval × group_ = 0.21, N.S.]. By contrast, the adult CS– LD, MD, and HD did not differ in their number of active responses for alcohol [*F*_(2, 38)tone × group_ = 0.44, N.S.; *F*_(4, 76) tone × interval × group_ = 1.9, N.S.; *F*_(2, 38) group_ = 1.14, N.S.].

Analysis of the active responses during the conditioned suppression test (Figure 3) revealed a significant effect of CS [*F*_(1, 153) CS_ = 15.7, *P* < 0.001], which was dependent on the tone presentation [*F*_(1, 153) tone × CS_ = 28.9, *P* < 0.001] and the interval [*F*_(2, 281) tone × interval × CS_ = 6.4, *P* < 0.01]. Moreover, conditioned suppression of alcohol seeking was dependent on the age of onset, as evident from a significant interval × age group × group × CS interaction [*F*_(3.4, 258)_ = 3.1, *P* < 0.05] and a significant age group × group × CS interaction [*F*_(2, 153)_ = 3.4, *P* < 0.05]. A similar pattern emerged after analysis of the data for the latency to the first response. There was an overall effect of CS [*F*_(1, 153) CS_ = 14, *P* < 0.001], which was dependent on presentation of the tone [*F*_(1, 153) tone × CS_ = 7.5, *P* < 0.01] and the interval [*F*_(2, 306) interval × CS_ = 8.1, *P* < 0.001]. Moreover, conditioned suppression was dependent on age of onset and group, in an interval and CS dependent manner [*F*_(4, 306) interval × age × group × CS_ = 2.8, *P* < 0.05] and there was a significant tone x interval x age group x group interaction [*F*_(2, 306)_ = 3.1, *P* < 0.05]. To further explore the differences between the groups, we analyzed the data separately for the LD, MD, and HD in the two age groups.

#### Adolescent onset

For the adolescent-onset LD (Figure 3A), there was no overall difference in responding between the CS– and CS+ groups [*F*_(1, 26) CS_ = 0.089, N.S], but the number of active lever presses in the CS+ and CS– rats was different when tone presentation was taken into account [*F*_(1, 26) tone × CS_ = 5.2, *P* < 0.05]. *Post-hoc* pairwise comparisons revealed no significant differences between the CS– and CS+ animals for any of the intervals. For the MD (see Figure 3B), the CS+ group showed a near-significant reduction in the number of active lever presses compared to CS– controls [*F*_(1, 26) CS_ = 3.6, *P* = 0.067], which was dependent on the tone presentation [*F*_(1, 26) tone × CS_ = 10.8, *P* < 0.01]. *Post-hoc* pairwise comparisons showed reduced responding in the CS+ group during the first tone ON interval. The HD group (Figure 3C) showed significant conditioned suppression, in that overall active responding during the conditioned suppression test was lower in the CS+ compared to the CS– animals [*F*_(1, 26)CS_ = 5.6, *P* < 0.05], independent of tone presentation [*F*_(1, 26) tone × CS_ = 2.2, N.S.]. *Post-hoc* pairwise comparisons showed decreased responding in the CS+ HD group during the first two tone ON intervals and the second tone OFF interval.

In agreement with the active response data, separate analyses of the latency data confirmed that adolescent-onset LD did not show suppression of alcohol seeking behavior, as evident from a lack of an overall difference between CS– and CS+ rats [Figure 4A; *F*_(1, 26) CS_ = 0.98, N.S.; *F*_(1, 26)tone × CS_ = 0.4, N.S.]. The MD and HD CS– and CS+ animals also did not differ in their latency to respond during the conditioned suppression test [MD: Figure 4B, *F*_(1, 26) CS_ = 3.4, N.S.; *F*_(1, 26) tone × CS_ = 3.4, N.S.; HD: Figure 4C, *F*_(1, 26) CS_ = 0.5, N.S.; *F*_(1, 26) tone × CS_ = 0.01, N.S.].

#### Adult onset

When comparing CS– and CS+ groups, only the LD animals showed conditioned suppression of alcohol seeking behavior. This was evident from an reduction in the number of active responses by the CS+ LD animals compared to the CS– controls [*F*_(1, 26) CS_ = 9.3, *P* < 0.01], which was dependent on tone presentation [*F*_(1, 26) tone × CS_ = 17.0, *P* < 0.001]. *Post-hoc* pairwise comparisons for the LD (Figure 3D) showed reduced alcohol seeking during all tone ON intervals and during the first tone OFF interval. By contrast, the MD did not show significant conditioned suppression of alcohol seeking [Figure 3E; *F*_(1, 24) CS_ = 0.0, N.S.; *F*_(1, 24) tone × CS_ = 2.2, N.S.]. Analysis of the active seeking responses for the HD (Figure 3F) showed no significant CS effect [*F*_(1, 25) CS_ = 2.7, *P* < 0.05; *F*_(1, 25) tone × CS_ = 2.9, N.S.].

Separate analyses of the latency data revealed that the adult-onset LD rats displayed a suppression of alcohol seeking when confronted with the shock-paired tone (Figure 4D) in that the latency to respond was greater for the CS+ compared to the CS– rats [*F*_(1, 26) CS_ = 7.7, *P* < 0.01; *F*_(2, 52) tone × CS_ = 2.4, N.S.]. By contrast, the adult-onset MD and HD rats did not show an altered latency to respond [MD: Figure 4E, *F*_(1, 24) CS_ = 2.1, N.S.; *F*_(1, 24) tone × CS_ = 0.8, N.S.; HD: Figure 4F, *F*_(1, 25) CS_ = 2.6, N.S.; *F*_(1, 25) tone × CS_ = 3.7, N.S.].

### Conditioned freezing behavior

**Figure 5:**
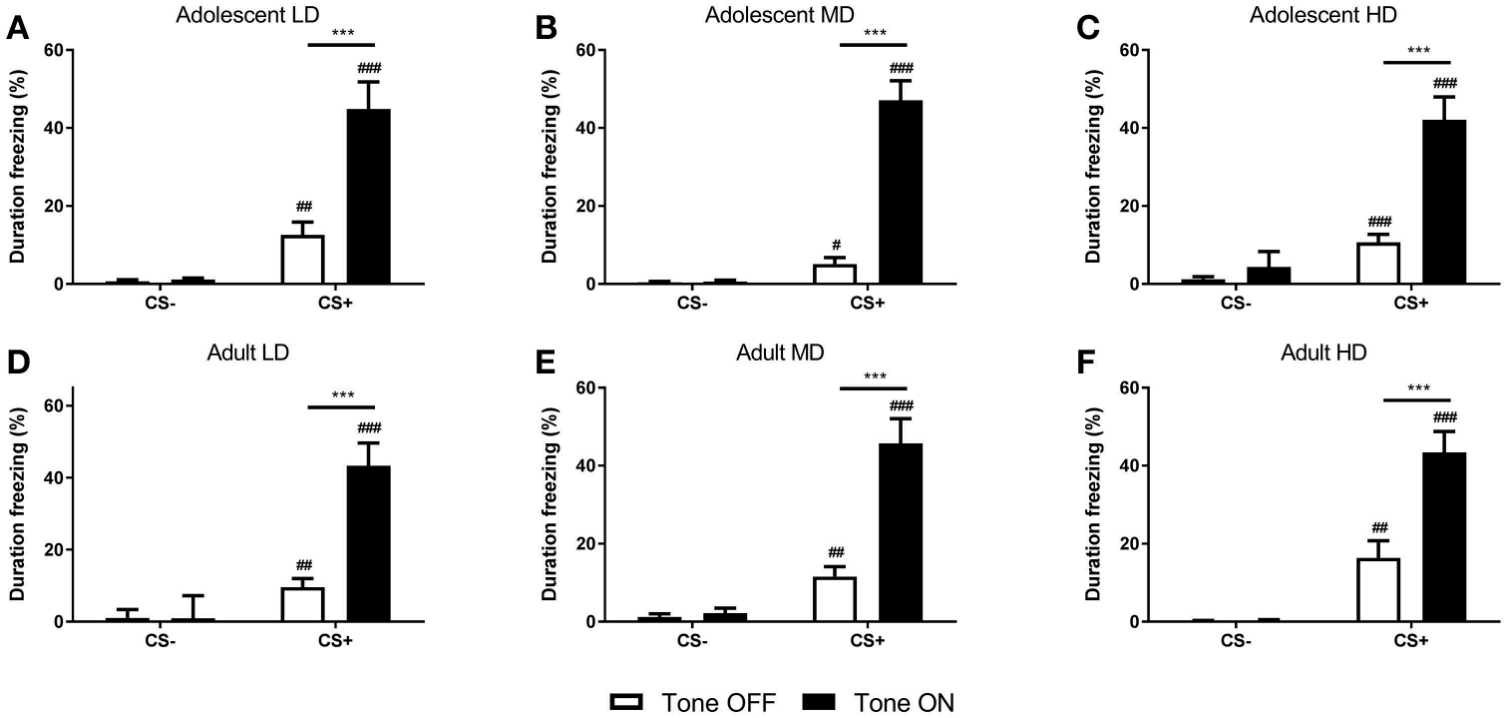
Freezing behavior in adolescent-onset low (LD; **A**), medium (MD; **B**), and high (HD; **C**) alcohol drinking rats and adult-onset LD **(D)**, MD **(E)**, and HD **(F)**. Shown is the duration of freezing in the conditioning chamber, expressed as a percentage of the 2 min prior to (Tone OFF) and during presentation of the footshock-associated CS+ (Tone ON) period. The conditioned rats (CS+) showed significant context- and CS-induced freezing, while the respective CS– control groups did not. This was comparable for all age and alcohol drinking groups. Data are presented as mean + SEM. ^#^, ^*##*^, ^*###*^Significant difference between CS– and CS+ groups (*post-hoc* pairwise comparisons, *P* < 0.05, *P* < 0.01, and *P* < 0.001, respectively). ***Significant difference between tone ON and tone OFF periods (*post-hoc* pairwise comparisons, *P* < 0.001).

After the conditioned suppression test, the rats were re-conditioned and conditioned freezing was determined. Analysis of freezing behavior of the rats during 2 min before (no-tone) and during CS (tone) presentation revealed that the time spent freezing was higher in CS+ rats compared to the CS– control animals [*F*_(1, 152) CS_ = 272, *P* < 0.001] (Figure 5). Moreover, conditioned freezing behavior was increased upon presentation of the footshock CS+ [*F*_(1, 152) tone × CS_ = 192, *P* < 0.001]. The degree of freezing behavior was comparable across age and groups [*F*_(2, 152) tone × age group × group × CS_ = 0.44, N.S.; *F*_(1, 152) tone × age group × CS_ = 0.33, N.S.; *F*_(2, 152) tone × group × CS_ = 1.5, N.S.; *F*_(2, 152) age group × group × CS_ = 0.57, N.S.].

## Discussion

The consumption of alcohol and the development of AUD often starts during adolescence. However, the relative impact of alcohol exposure during adolescence, as compared to adulthood, on the emergence of AUD later in life is poorly understood. Therefore, in the present study, we compared alcohol intake and control over alcohol seeking in animals that started to consume alcohol in either adolescence or adulthood. Rats with an adolescent onset of alcohol drinking consumed less alcohol than rats that started to drink alcohol in adulthood. In addition, we found that control over alcohol seeking was differentially related to the level of alcohol intake in the two age groups. In adult-onset rats, conditioned suppression of alcohol seeking was observed in the LD, but not the MD or HD, i.e., being negatively related to levels of alcohol intake. In the adolescent-onset group the MD and HD, that showed higher levels of alcohol seeking compared to the LD, showed significantly reduced alcohol seeking upon presentation of conditioned aversive stimuli. These findings indicate a complex relationship between age of alcohol drinking onset, level of alcohol intake, and control over alcohol seeking.

### Alcohol intake in adolescent and adult rats

We observed a modest but significant difference in alcohol intake when comparing the two age groups. The adolescent-onset rats consumed less alcohol when compared to adult rats. This was most pronounced during the second month of alcohol intake, when rats had 24 h access to alcohol. Perhaps the age differences were more prominent during this phase of the experiment because alcohol consumption levels are higher when access time to alcohol is extended, as we have shown previously (39). Other studies that directly compared alcohol intake in age groups that were tested in parallel, comparable to our approach, showed that adolescent rats consumed equal or lower amounts of alcohol than rats with an adult onset of alcohol intake (24, 28, 51), although adolescent rats have been shown to respond more for alcohol in an operant task (30). The adolescent rats also showed a lower preference for alcohol and the adolescents overall consumed more water compared to the adult rats, which is in line with previous reports (28, 51). Taken together, our findings show that adolescent rats do not drink more alcohol than their adult counterparts. These findings are in apparent contrast to the human literature, which shows that alcohol use peaks during adolescence (7). Although speculative, it is likely that this discrepancy is related to the housing conditions. While adolescent alcohol use in humans is thought to be driven in part by the social environment, for example by social attunement (8), the rats in this study were individually housed, precluding a potential impact of social factors.

In apparent contrast to our present, and other observations (24, 28, 51), it has previously been suggested that alcohol intake in adolescence promotes the development of AUD-like behavior. This was based on studies that reported augmented alcohol intake or operant responding for alcohol in adult rats that were exposed to alcohol during adolescence (20–24). However, it should be borne in mind that in these experiments, alcohol intake after adolescent pre-exposure was compared to adolescent non-exposure to alcohol. Moreover, alcohol intake or operant responding was determined after an abstinence period of 3–6 weeks. It is therefore possible that the increase in alcohol intake after adolescent alcohol exposure reflects an alcohol deprivation effect, i.e., an increase in alcohol intake after an abstinence phase [e.g., (52–60)], rather than an age-dependent risk for AUD. In fact, Siegmund et al. (28) have shown that the alcohol deprivation effect is comparable in adolescent- and adult-onset drinking rats. Therefore, reports of adolescent alcohol exposure-induced changes in alcohol intake may reflect the impact of adolescent exposure to alcohol, but may also mirror the effect of an alcohol deprivation phase.

### Age of drinking onset influences control over alcohol seeking

To determine the impact of adolescent vs. adult onset of alcohol exposure on the individuals' risk to develop AUD-like behavior, we directly compared two age groups, i.e., commencing alcohol intake at PND 42 vs. PND 77, in a parallel design. We assessed the degree of control over alcohol seeking in these age groups using a conditioned suppression test. In this test, animals are exposed to a conflict situation where they can seek alcohol but at the same time are confronted with a warning sign that has previously been associated with unpredictable mild electric footshocks. Continued substance seeking under these conditions is interpreted as loss of control over alcohol seeking (40, 45, 47). The adult rats displayed a similar pattern to what we have previously reported (40), in that adult LD showed a clear suppression of alcohol seeking, while MD and HD did not. This was apparent both from the active response data and from the latency data, where LD CS+ rats show reduced lever pressing and a longer latency to press upon presentation of the aversive stimulus. These differences between the groups were not due to differences in baseline responding, which were comparable for the LD, MD, and HD CS– groups. By contrast, the adolescent MD and HD, but not the LD rats showed significant conditioned suppression of alcohol seeking. Importantly, the differences in alcohol intake between adolescents and adults were only modest. Moreover, adolescent-onset rats consumed less alcohol compared to adult-onset rats, but adolescent HD rats consumed more alcohol than adult MD, that did not display suppression of alcohol seeking. Therefore, the suppression of alcohol seeking in adolescent MD and HD cannot be explained by low levels of preceding alcohol consumption. The remarkable absence of conditioned suppression in the adolescent-onset LD could indicate reduced control over alcohol use in rats that consume low amounts of alcohol. However, this is not likely, given that our previous studies demonstrate more control over alcohol use in low alcohol drinking animals, in comparison to their high alcohol drinking counterparts (39, 40). Rather, we think that the absence of conditioned suppression in the adolescent-onset LD reflects a floor-effect, due to the low baseline levels of responding in this group.

Taken together, these results indicate that whereas in adult-onset rats, control over alcohol seeking is inversely related to the level of preceding alcohol intake, adolescent-onset alcohol exposure less readily leads to loss of control over alcohol seeking. The neural and behavioral mechanisms underlying this age difference remain to be elucidated, but comparable findings have previously been reported. For example, Jeanblanc et al. (61) characterized rats, with or without a neonatal ventral hippocampus lesion (NVHL), that were exposed to low levels of alcohol during adolescence for the expression of AUD-like behavioral characteristics (61). The sham rats in this study did not develop AUD-like behavior, i.e., did not show greater motivation for alcohol, resistance to extinction or an increase in reinstatement of alcohol seeking. Moreover, Serlin and Torregrossa (30) have shown reduced habit formation in rats that were exposed to alcohol during adolescence. This latter observation may reflect a relative resilience to AUD, considering that habit formation is thought to contribute to the development of substance use disorders [e.g., (62)].

Our findings suggest that rats that are exposed to alcohol during adolescence are more flexible in the conditioned suppression task, as they adjust their alcohol seeking behavior in this conflict situation. It is tempting to speculate about the implications of adolescent alcohol exposure for the development of behavioral flexibility in general. Intermittent exposure to intoxicating levels of alcohol during adolescence has been shown to result in impaired performance in a reversal learning task in rats (63), but similar findings have been reported for adult rats (64). Therefore, high levels of alcohol may be detrimental for behavioral flexibility, independent of age. There is some evidence from human studies, which suggests that binge drinking during adolescence (13–18 years) does not impair the performance in executive tasks, while binge drinking during young adulthood (19–22 years) does (65), suggesting that the adolescent brain may compensate for alcohol intoxication to maintain behavioral flexibility. However, this age range only partly overlaps with early adolescent onset of alcohol use, i.e., before the age of 15, which has been shown to increase the likelihood for developing an AUD (9–16). Therefore, it remains unclear whether and how adolescent alcohol exposure alters behavioral flexibility, as a potential mechanism underlying the apparent adolescent resilience to loss of control over alcohol use. Future studies are necessary to fully understand the consequences of adolescent vs. adult alcohol exposure on control over behavior and the underlying neural mechanisms.

A limitation of the present study is the use of males only. Considering the gender differences in alcohol use and AUD, follow-up studies should be extended to determine the impact of age in females. A further limitation is that loss of control was determined when the rats reached adulthood and we have no data on adolescent behavior. This is inherent to the paradigms that we have used. Previously, we have shown that HD develop loss of control after 8 weeks of alcohol consumption (40), which is the exposure time to alcohol that was used here. In the present study, the adolescent-onset animals started to consume alcohol (for 8 consecutive weeks) when they were 6 weeks of age (PND 42) and were subsequently trained to respond for alcohol under fixed and random interval schedules of reinforcement for at least 7 consecutive weeks. Rats are considered adult from 8 to 10 weeks of age. Therefore, our adolescent-onset rats were adults when we assessed loss of control over alcohol seeking. To overcome this limitation, future studies should determine the duration of alcohol consumption after which rats develop resistance to conditioned suppression.

In conclusion, the present findings reveal a complex age-dependence of alcohol drinking and control over alcohol seeking, whereby adolescent rats consume less alcohol than adults. Moreover, while control over alcohol seeking in adult-onset rats is negatively related to preceding levels of alcohol intake, adolescent-onset rats appear to retain control over alcohol seeking, even after consuming high levels of alcohol. These findings support our hypothesis that rats are less susceptible to lose control over alcohol seeking when alcohol intake is initiated during adolescence as compared to adulthood. Future studies should be directed at understanding the underlying mechanisms of a possible, relative resilience to AUD in adolescents. This may inform prevention and treatment strategies for AUD, aimed at both the young and the adult.

## Author contributions

HL, JC, JK, and LV designed the study. ML and LJ performed the experiments. ML and HL analyzed the data. HL, JC, JK, and LV wrote the paper with input from all authors.

### Conflict of interest statement

The authors declare that the research was conducted in the absence of any commercial or financial relationships that could be construed as a potential conflict of interest.

## References

[1] Anderson P. Global use of alcohol, drugs and tobacco. Drug Alcohol Rev. (2006) 25:489–502. 10.1080/09595230600944446 17132569

[2] Rehm J Mathers C Popova S Thavorncharoensap M Teerawattananon Y Patra J. Global burden of disease and injury and economic cost attributable to alcohol use and alcohol-use disorders. Lancet (2009) 373:2223–33. 10.1016/S0140-6736(09)60746-7 19560604

[3] WHO RE: Global Status Report on Alcohol and Health. World Health Organization (2011).

[4] American Psychiatric Association Diagnostic and Statistical Manual of Mental Disorders. 5th ed. Washington, DC: American Psychiatric Association (2013).

[5] Squeglia LM Jacobus J Tapert SF. The effect of alcohol use on human adolescent brain structures and systems. Handb Clin Neurol. (2014) 125:501–10. 10.1016/B978-0-444-62619-6.00028-8 25307592 PMC4321715

[6] Crone EA Dahl RE. Understanding adolescence as a period of social-affective engagement and goal flexibility. Nat rev Neurosci. (2012) 13:636–50. 10.1038/nrn3313 22903221

[7] Dennis M Scott CK. Managing addiction as a chronic condition. Addict Sci Clin Pract. (2007) 4:45–55. 10.1151/ascp074145 18292710 PMC2797101

[8] Cousijn J Luijten M Feldstein Ewing SW Adolescent resilience to addiction: a social plasticity hypothesis. Lancet Child Adolesc Health (2018) 2:69–78. 10.1016/S2352-4642(17)30148-7 30169197 PMC6373770

[9] Grant BF Dawson DA. Age at onset of alcohol use and its association with DSM-IV alcohol abuse and dependence: results from the National Longitudinal alcohol epidemiologic survey. J Subst Abuse (1997) 9:103–10. 10.1016/S0899-3289(97)90009-2 9494942

[10] Pedersen W Skrondal A. Alcohol consumption debut: predictors and consequences. J Stud Alcohol (1998) 59:32–42. 10.15288/jsa.1998.59.32 9498313

[11] DeWit DJ Adlaf EM Offord DR Ogborne AC. Age at first alcohol use: a risk factor for the development of alcohol disorders. Am J Psychiatry (2000) 157:745–50. 10.1176/appi.ajp.157.5.745 10784467

[12] Pitkanen T Lyyra AL Pulkkinen L. Age of onset of drinking and the use of alcohol in adulthood: a follow-up study from age 8-42 for females and males. Addiction (2005) 100:652–61. 10.1111/j.1360-0443.2005.01053.x 15847623

[13] Dawson DA Goldstein RB Chou SP Ruan WJ Grant BF. Age at first drink and the first incidence of adult-onset DSM-IV alcohol use disorders. Alcohol Clin Exp Res. (2008) 32:2149–60. 10.1111/j.1530-0277.2008.00806.x 18828796 PMC2760820

[14] Sartor CE Lynskey MT Bucholz KK Madden PA Martin NG Heath AC. Timing of first alcohol use and alcohol dependence: evidence of common genetic influences. Addiction (2009) 104:1512–8. 10.1111/j.1360-0443.2009.02648.x 19686520 PMC2741422

[15] Liang W Chikritzhs T. Age at first use of alcohol and risk of heavy alcohol use: a population-based study. Biomed Res Int. (2013) 2013:721761. 10.1155/2013/721761 24471139 PMC3891545

[16] Asbridge M Cartwright J Wilson K Langille D. Age at first drink, experiences of drunkenness, and alcohol-related problems in Canadian youth: Is early onset bad if you are a moderate drinker? J Stud Alcohol Drugs (2016) 77:974–9. 10.15288/jsad.2016.77.974 27797700

[17] McGue M Iacono WG Legrand LN Malone S Elkins I Origins and consequences of age at first drink. I. Associations with substance-use disorders, disinhibitory behavior and psychopathology, and P3 amplitude. Alcohol Clin Exp Res. (2001) 25:1156–65. 10.1111/j.1530-0277.2001.tb02330.x 11505047

[18] Maimaris W McCambridge J. Age of first drinking and adult alcohol problems: systematic review of prospective cohort studies. J Epidemiol Commun Health (2014) 68:268–74. 10.1136/jech-2013-203402 24249000 PMC4158030

[19] Kuntsche E Rossow I Engels R Kuntsche S. Is 'age at first drink' a useful concept in alcohol research and prevention? We doubt that. Addiction (2016) 111:957–65. 10.1111/add.12980 26147610

[20] Alaux-Cantin S Warnault V Legastelois R Botia B Pierrefiche O Vilpoux C. Alcohol intoxications during adolescence increase motivation for alcohol in adult rats and induce neuroadaptations in the nucleus accumbens. Neuropharmacology (2013) 67:521–31. 10.1016/j.neuropharm.2012.12.007 23287538

[21] Milivojevic V Covault J Alcohol exposure during late adolescence increases drinking in adult Wistar rats, an effect that is not reduced by finasteride. Alcohol Alcohol. (2013) 48:28–38. 10.1093/alcalc/ags105 22997410 PMC3523383

[22] O'Tousa DS Matson LM Grahame NJ. Effects of intoxicating free-choice alcohol consumption during adolescence on drinking and impulsivity during adulthood in selectively bred high-alcohol preferring mice. Alcohol Clini Exp Res. (2013) 37:141–9. 10.1111/j.1530-0277.2012.01857.x 22725646 PMC4259273

[23] Pandey SC Sakharkar AJ Tang L Zhang H. Potential role of adolescent alcohol exposure-induced amygdaloid histone modifications in anxiety and alcohol intake during adulthood. Neurobiol Dis. (2015) 82:607–19. 10.1016/j.nbd.2015.03.019 25814047 PMC4581895

[24] Amodeo LR Kneiber D Wills DN Ehlers CL. Alcohol drinking during adolescence increases consumptive responses to alcohol in adulthood in wistar rats. Alcohol (2017) 59:43–51. 10.1016/j.alcohol.2016.12.002 28187948 PMC5358807

[25] Varlinskaya EI Kim EU Spear LP. Chronic intermittent ethanol exposure during adolescence: effects on stress-induced social alterations and social drinking in adulthood. Brain Res. (2017) 1654:145–56. 10.1016/j.brainres.2016.03.050 27048754 PMC5047849

[26] Gilpin NW Karanikas CA Richardson HN. Adolescent binge drinking leads to changes in alcohol drinking, anxiety, and amygdalar corticotropin releasing factor cells in adulthood in male rats. PLoS ONE (2012) 7:e31466. 10.1371/journal.pone.0031466 22347484 PMC3275622

[27] Schramm-Sapyta NL DiFeliceantonio AG Foscue E Glowacz S Haseeb N Wang N. Aversive effects of ethanol in adolescent versus adult rats: potential causes and implication for future drinking. Alcohol. Clin. Exp Res. (2010) 34:2061–9. 10.1111/j.1530-0277.2010.01302.x 20860614 PMC2988872

[28] Siegmund S Vengeliene V Singer MV Spanagel R. Influence of age at drinking onset on long-term ethanol self-administration with deprivation and stress phases. Alcohol. Clin. Exp Res. (2005) 29:1139–45. 10.1097/01.ALC.0000171928.40418.46 16046868

[29] Vetter CS Doremus-Fitzwater TL Spear LP. Time course of elevated ethanol intake in adolescent relative to adult rats under continuous, voluntary-access conditions. Alcohol Clin Exp Res. (2007) 31:1159–68. 10.1111/j.1530-0277.2007.00417.x 17511750 PMC2094127

[30] Serlin H Torregrossa MM. Adolescent rats are resistant to forming ethanol seeking habits. Dev Cogn Neurosci. (2015) 16:183–90. 10.1016/j.dcn.2014.12.002 25575668 PMC4480209

[31] Nasrallah NA Yang TW Bernstein IL. Long-term risk preference and suboptimal decision making following adolescent alcohol use. Proc Nat Acad Sci USA. (2009) 106:17600–4. 10.1073/pnas.0906629106 19805186 PMC2765167

[32] Nasrallah NA Clark JJ Collins AL Akers CA Phillips PE Bernstein IL Risk preference following adolescent alcohol use is associated with corrupted encoding of costs but not rewards by mesolimbic dopamine. Proc Nat Acad Sci USA. (2011) 108:5466–71. 10.1073/pnas.1017732108 21402915 PMC3069180

[33] Schindler AG Tsutsui KT Clark JJ Chronic alcohol intake during adolescence, but not adulthood, promotes persistent deficits in risk-based decision making. Alcohol Clin Exp Res. (2014) 38:1622–9. 10.1111/acer.12404 24689661 PMC4047126

[34] Miller KM Risher ML Acheson SK Darlow M Sexton HG Schramm-Sapyta N. Behavioral inefficiency on a risky decision-making task in adulthood after adolescent intermittent ethanol exposure in rats. Sci Rep. (2017) 7:4680. 10.1038/s41598-017-04704-7 28680108 PMC5498633

[35] McClory AJ Spear LP. effects of ethanol exposure during adolescence or in adulthood on pavlovian conditioned approach in sprague-dawley rats. Alcohol (2014) 48:755–63. 10.1016/j.alcohol.2014.05.006 25449366 PMC4254554

[36] Spoelder M Tsutsui KT Lesscher HMB Vanderschuren LJMJ Clark JJ. Adolescent alcohol exposure amplifies the incentive value of reward-predictive cues through potentiation of phasic dopamine signaling. Neuropsychopharmacology (2015) 40:2873–85. 10.1038/npp.2015.139 25971592 PMC4864623

[37] Spear LP. Adolescent alcohol exposure: are there separable vulnerable periods within adolescence? Physiol Behav. (2015) 148:122–30. 10.1016/j.physbeh.2015.01.027 25624108 PMC4484315

[38] Crews FT Vetreno RP Broadwater MA Robinson DL. Adolescent alcohol exposure persistently impacts adult neurobiology and behavior. Pharmacol Rev. (2016) 68:1074–109. 10.1124/pr.115.012138 27677720 PMC5050442

[39] Spoelder M Vanderschuren LJMJ Lesscher HMB. Individual variation in alcohol intake predicts reinforcement, motivation, and compulsive alcohol use in rats. Alcohol Clin Exp Res. (2015b) 39:2427–37. 10.1111/acer.12891 26745576

[40] Spoelder M Pol S Janssen BSG Baars AM Vanderschuren LJMJ Lesscher HMB. Loss of control over alcohol seeking in rats depends on individual vulnerability and duration of alcohol consumption experience. Behav Pharmacol. (2017) 28:334–44. 10.1097/FBP.0000000000000304 28418943

[41] Vetter-O'Hagen CS Spear LP. The effects of gonadectomy on sex- and age-typical responses to novelty and ethanol-induced social inhibition in adult male and female Sprague-Dawley rats. Behav Brain Res. (2012) 227:224–32. 10.1016/j.bbr.2011.10.023 22036699 PMC3242866

[42] Whitaker LR Degoulet M Morikawa H. Social deprivation enhances VTA synaptic plasticity and drug-induced contextual learning. Neuron (2013) 77:335–45. 10.1016/j.neuron.2012.11.022 23352169 PMC3559005

[43] Baarendse PJ Limpens JH Vanderschuren LJMJ. Disrupted social development enhances the motivation for cocaine in rats. Psychopharmacology (2014) 231:1695–704. 10.1007/s00213-013-3362-8 24311358 PMC3969396

[44] Lesscher HMB Spoelder M Rotte MD Janssen MJ Hesseling P Lozeman-van 't Klooster JG. Early social isolation augments alcohol consumption in rats. Behav Pharmacol. (2015) 26:673–80. 10.1097/FBP.0000000000000165 26325660

[45] Vanderschuren LJMJ Everitt BJ. Drug seeking becomes compulsive after prolonged cocaine self-administration. Science (2004) 305:1017–9. 10.1126/science.1098975 15310907

[46] Limpens JHW Damsteegt R Broekhoven MH Vanderschuren LJMJ Pharmacological inactivation of the prelimbic cortex emulates compulsive cocaine seeking in rats. Brain Res. (2014) 1628:210–8. 10.1016/j.brainres.2014.10.045 25451128

[47] Limpens JHW Schut EHS Vanderschuren LJMJ. Using conditioned suppression to investigate compulsive drug seeking in rats. Drug Alcohol Depend. (2014) 142:314–24. 10.1016/j.drugalcdep.2014.06.037 25060961

[48] Blanchard RJ Blanchard DC. Passive and active reactions to fear-eliciting stimuli. J. Comp Physiol Psychol. (1969) 68:129–35. 10.1037/h0027676 5793861

[49] Bouton ME Bolles RC Conditioned fear assessed by freezing and by the suppression of three different baselines. Anim Learn Behav. (1980) 8:429–34. 10.3758/BF03199629

[50] LeDoux JE Sakaguchi A Reis DJ. Subcortical efferent projections of the medial geniculate nucleus mediate emotional responses conditioned to acoustic stimuli. J Neurosci. (1984) 4:683–98. 10.1523/JNEUROSCI.04-03-00683.1984 6707732 PMC6564820

[51] Bell RL Rodd ZA Sable HJ Schultz JA Hsu CC Lumeng L. Daily patterns of ethanol drinking in peri-adolescent and adult alcohol-preferring (P) rats. Pharmacol Biochem Behav. (2006) 83:35–46. 10.1016/j.pbb.2005.12.004 16442608

[52] Spanagel R Hölter SM. Long-term alcohol self-administration with repeated alcohol deprivation phases: an animal model of alcoholism? Alcohol Alcohol. (1999) 34:231–43. 10344783 10.1093/alcalc/34.2.231

[53] Rodd-Henricks ZA McKinzie DL Murphy JM McBride WJ Lumeng L Li TK. The expression of an alcohol deprivation effect in the high-alcohol-drinking replicate rat lines is dependent on repeated deprivations. Alcohol Clin Exp Res. (2000) 24:747–53. 10.1111/j.1530-0277.2000.tb02051 10888060

[54] Rodd-Henricks ZA McKinzie DL Shaikh SR Murphy JM McBride WJ Lumeng L. Alcohol deprivation effect is prolonged in the alcohol preferring (P) rat after repeated deprivations. Alcohol Clin Exp Res. (2000) 24:8–16. 10.1111/j.1530-0277.2000.tb04546.x 10656186

[55] Spanagel R Hölter SM. Pharmacological validation of a new animal model of alcoholism. J Neural Transm. (2000) 107:669–80. 10.1007/s007020070068 10943907

[56] Serra S Brunetti G Pani M Vacca G Carai MA Gessa GL. Blockade by the cannabinoid CB(1) receptor antagonist, SR 141716, of alcohol deprivation effect in alcohol-preferring rats. Eur J Pharmacol. (2002) 443:95–7. 10.1016/S0014-2999(02)01594-7 12044797

[57] Colombo G Serra S Brunetti G Vacca G Carai MA Gessa GL. Suppression by baclofen of alcohol deprivation effect in Sardinian alcohol-preferring (sP) rats. Drug Alcohol Depend. (2003) 70:105–8. 10.1016/S0376-8716(02)00333-2 12681531

[58] McBride WJ Rodd ZA Bell RL Lumeng L Li TK. The alcohol-preferring (P) and high-alcohol-drinking (HAD) rats–animal models of alcoholism. Alcohol (2014) 48:209–15. 10.1016/j.alcohol.2013.09.044 24268381 PMC4006324

[59] Momeni S Roman E. Subgroup-dependent effects of voluntary alcohol intake on behavioral profiles in outbred Wistar rats. Behav Brain Res. (2014) 275:288–96. 10.1016/j.bbr.2014.08.058 25200519

[60] Vengeliene V Bilbao A Spanagel R. The alcohol deprivation effect model for studying relapse behavior: a comparison between rats and mice. Alcohol (2014) 48:313–20. 10.1016/j.alcohol.2014.03.002 24811155

[61] Jeanblanc J Balguerie K Coune F Legastelois R Jeanblanc V Naassila M. Light alcohol intake during adolescence induces alcohol addiction in a neurodevelopmental model of schizophrenia. Addict Biol. (2015) 20:490–9. 10.1111/adb.12146 24725220

[62] O'Tousa D Grahame N. Habit formation: implications for alcoholism research. Alcohol (2014) 48:327–35. 10.1016/j.alcohol.2014.02.004 24835007 PMC4096986

[63] Fernandez GM Lew BJ Vedder LC Savage LM. Chronic intermittent ethanol exposure leads to alterations in brain-derived neurotrophic factor within the frontal cortex and impaired behavioral flexibility in both adolescent and adult rats. Neuroscience (2017) 348:324–34. 10.1016/j.neuroscience.2017.02.045 28257889 PMC5458357

[64] Fernandez GM Stewart WN Savage LM. Chronic drinking during adolescence predisposes the adult rat for continued heavy drinking: neurotrophin and behavioral adaptation after long-term, continuous ethanol exposure. PLoS ONE (2016) 11:e0149987. 10.1371/journal.pone.0149987 26930631 PMC4773001

[65] Gil-Hernandez S Mateos P Porras C Garcia-Gomez R Navarro E Garcia-Moreno LM. Alcohol Binge Drinking and Executive Functioning during Adolescent Brain Development. Front Psychol. (2017) 8:1638. 10.3389/fpsyg.2017.01638 29046650 PMC5632721

